# Health Care Professionals’ Perspectives on Using eHealth Tools in Advanced Home Care: Qualitative Interview Study

**DOI:** 10.2196/60582

**Published:** 2025-03-24

**Authors:** Eric Vincent Rivas, Ulf Lesley, Nadia Davoody

**Affiliations:** 1 Health Informatics Centre Department of Learning, Informatics, Management and Ethics Karolinska Institutet Stockholm Sweden

**Keywords:** eHealth, mobile health, mHealth, advanced home care, content analysis, nurse, staff-patient relationship, aging population, patient engagement, personalized care, patient experience

## Abstract

**Background:**

The rising demand for advanced home care services, driven by an aging population and the preference for aging in place, presents both challenges and opportunities. While advanced home care can improve cost-effectiveness and patient outcomes, gaps remain in understanding how eHealth technologies can optimize these services. eHealth tools have the potential to offer personalized, coordinated care that increases patient engagement. However, research exploring health care professionals’ (HCPs) perspectives on the use of eHealth tools in advanced home care and their impact on the HCP-patient relationship is limited.

**Objective:**

This study aims to explore HCPs’ perspectives on using eHealth tools in advanced home care and these tools’ impact on HCP-patient relationships.

**Methods:**

In total, 20 HCPs from 9 clinics specializing in advanced home care were interviewed using semistructured interviews. The discussions focused on their experiences with 2 eHealth tools: a mobile documentation tool and a mobile preconsultation form. The data were analyzed using content analysis to identify recurring themes.

**Results:**

The data analysis identified one main theme: optimizing health care with eHealth; that is, enhancing care delivery and overcoming challenges for future health care. Two subthemes emerged: (1) enhancing care delivery, collaboration, and overcoming adoption barriers and (2) streamlining implementation and advancing eHealth tools for future health care delivery. Five categories were also identified: (1) positive experiences and benefits, (2) interactions between HCPs and patients, (3) challenges and difficulties with eHealth tools, (4) integration into the daily workflow, and (5) future directions. Most HCPs expressed positive experiences with the mobile documentation tool, highlighting improved efficiency, documentation quality, and patient safety. While all found the mobile preconsultation form beneficial, patient-related factors limited its utility. Regarding HCP-patient relationships, interactions with patients remained unchanged with the implementation of both tools. HCPs successfully maintained their interpersonal skills and patient-centered approach while integrating eHealth tools into their practice. The tools allowed more focused, in-depth discussions, enhancing patient engagement without affecting relationships. Difficulties with the tools originated from tool-related issues, organizational challenges, or patient-related complexities, occasionally affecting the time available for direct patient interaction.

**Conclusions:**

The study underscores the importance of eHealth tools in enhancing advanced home care while maintaining the HCP-patient relationship. While eHealth tools modify care delivery techniques, they do not impact the core dynamics of the relationships between HCPs and patients. While most of the HCPs in the study had a positive attitude toward using the eHealth tools, understanding the challenges they encounter is crucial for improving user acceptance and success in implementation. Future development should focus on features that not only improve efficiency but also actively enhance HCP-patient relationships, such as facilitating more meaningful interactions and supporting personalized care in the advanced home care setting.

## Introduction

### Advanced Home Care

Advanced home care, also known as advanced health care in the home, involves providing medical care or treatment directly at a patient’s home, serving as an alternative care to inpatient care. The World Health Organization defines home care as “an array of health and social support services provided to clients in their residence. Such coordinated services may prevent, delay, or be a substitute for temporary or long-term institutional care” [1]. According to a study by Barakat et al [2], the components of home care include preventive actions and assessments, postdischarge actions, and evaluations, with objectives focused on enhancing or maintaining the quality of life, optimizing functional health status, and promoting independence. The composition of health care professionals (HCPs) providing home care varies depending on the patient’s needs and the extent of services they require. This team may involve various HCPs, such as physicians, nurses, assistant nurses, physical therapists, occupational therapists, speech-language pathologists, home health aides, home infusion nurses (specialized nurses who administer intravenous medications in patients’ homes), hospice caregivers, and medical social workers [3]. Patients receiving home care may have a wide range of health conditions and diseases, including chronic conditions, such as diabetes, hypertension, and heart disease; neurological conditions, such as Parkinson disease, dementia, and stroke; respiratory conditions, such as chronic obstructive pulmonary disease, asthma, and sleep apnea; cancer; individuals requiring wound care; and those in need of palliative care [3].

The increasing demand for home care services is driven not only by an aging population but also by the growing preference among many individuals to age in place, remaining in their own homes. The primary goal of advanced home care is to enhance patients’ quality of life and clinical outcomes while simultaneously reducing health care costs and hospital readmissions [4].

### Use of Electronic Health in Advanced Home Care

Organizational suppliers of health care are increasingly delivering medical care directly to patients’ homes, which has created a growing reliance on medical technologies and eHealth tools [1] to support health care staff in managing not only older people care but also palliative and end-of-life care and patients with multiple comorbidities or complex diseases. The increased presence of eHealth tools also highlights the importance of considering the competencies and requirements of HCPs for using eHealth tools in older people care [2].

eHealth’s vital role in advanced home care includes using telehealth, remote patient monitoring, and mobile health apps to deliver health care services to patients with complex medical needs in their homes [5]. eHealth tools facilitate communication between patients and HCPs, support medication management, monitor vital signs and symptoms, and offer educational resources to patients and caregivers. Furthermore, integrating eHealth into advanced home care can also enable organizational suppliers of health to deliver personalized and coordinated care, increase patient engagement and self-management, and improve overall patient satisfaction [2].

In Sweden, the national policy emphasizes home assistance and home care over institutionalized care, with various programs supporting long-term care for older people, assistance for people with disabilities, end-of-life care, palliative care in hospitals or hospices, and advanced palliative home care administered by municipalities. In 2017, home care met 72% of long-term care needs, while institutions served the remaining 28% [6]. Given the substantial reliance on home care for health care delivery, there is a significant need for resources, making eHealth services, such as those mentioned earlier, highly beneficial [7].

A study by Rydenfält et al [8] outlined the implementation of eHealth services in Swedish home care nursing, listing national patient summaries, mobile documentation, digital locks, digital medical lists, digital security alarms, and camera supervision as the most commonly used services. Organizational suppliers of health care had implemented mobile documentation most widely among these services at the time of the study, with plans for its continued use in the future for home care nursing.

### Traditional Documentation

Health care staff have long used traditional methods, such as pen-and-paper notes, to document patient encounters, including observations, assessments, signs, symptoms, and communications during clinic consultations and home care visits. While some remnants of this traditional documentation method may persist in current practices, health care organizations have adopted information and communication technology solutions [9] and notable mobile communications [10] to address the inherent challenges of traditional documentation. Traditional documentation methods occasionally lead HCPs to omit crucial information inadvertently, record data inaccurately, produce illegible handwriting, and misunderstand patients’ accounts, while requiring significant time for documentation [11].

### Patient Experiences With eHealth

Researchers have extensively explored patient experiences with eHealth, particularly in home and palliative care settings, and have revealed a range of benefits and challenges. Steindal et al [3] found that telehealth applications in palliative home care enhance access to HCPs, helping patients feel more secure and safe. Similarly, Widberg et al [4] highlighted that eHealth applications facilitate better communication between patients and HCPs in palliative care. Karlsen et al [12] focused on older adults’ use of telecare in home care services and emphasized their desire to age in place. These studies show that patients view technology as a valuable tool to achieve this goal, although some resist adopting new technologies, struggle with digital proficiency, and worry about privacy and security [5].

Older patients face notable challenges when using eHealth tools. These include their preference for direct contact with HCPs, age-related cognitive decline, the stigmatization of telemonitoring devices, and the potential loss of social interactions, which are essential for well-being [5,13]. Experts emphasize the importance of balancing in-person care with eHealth use for certain populations, such as older patients with chronic conditions. Despite these obstacles, studies highlight that eHealth improves care accessibility and patient autonomy [3,5,12].

Specific eHealth applications directly benefit patients. For instance, an eHealth app tested in home care settings allowed patients to report health concerns and increased their sense of security [10] by reducing the frequency of phone calls to nurses, enabling them to prioritize care based on the app’s alerts. However, frequent reporting through the app led HCPs to perceive minor health issues as significant, which increased their workload [10]. These findings illustrate the nuanced impact of eHealth on patient experiences and underline the need to carefully address patients’ needs and contexts.

### The Acceptance and Perspectives of HCPs on eHealth

HCPs play a pivotal role in adopting and successfully implementing eHealth solutions, as their acceptance and perspectives directly influence outcomes. Li et al [14] identified key factors that affect HCPs’ acceptance of eHealth. These factors include HCP characteristics, voluntariness of use, performance and effort expectancy, and how organizational conditions either support or hinder eHealth adoption. HCPs emphasize patient autonomy, personalization, and continuity of professional support as facilitators of eHealth adoption, while they note advanced patient age as a primary barrier [15].

Qualitative studies offer additional insights into HCPs’ attitudes toward eHealth. In an interview study, general practitioners expressed positive attitudes toward eHealth as a means to promote healthy lifestyles for patients and themselves [16]. They showed confidence in transitioning from traditional paper-based approaches to digital solutions, particularly those incorporating patient-reported outcome measures. Similarly, Das et al [17] examined the impact of an eHealth portal on HCP-patient interactions and reported that HCPs value the portal for its ability to provide comprehensive patient information, foster accountability, and serve as a clinical tool. However, they also identified organizational challenges, such as a lack of incentives and time constraints, which hinder eHealth integration.

Understanding HCPs’ experiences and perceptions is essential for optimizing eHealth tools. While eHealth enhances health care quality, efficiency, and accessibility, its success depends on addressing the barriers that HCPs face. Targeted training and strategies to overcome organizational constraints can improve HCP satisfaction and ensure seamless integration of eHealth into clinical practice [18].

### Aim of the Study

Despite extensive research in eHealth, there remains a limited understanding of how eHealth tools affect the relationship between HCPs and patients in advanced home care settings. While some studies have explored patient perspectives, fewer researchers have focused on HCPs’ experiences and perceptions.

The aim of this study is to explore HCPs’ perspectives on using eHealth tools in advanced home care and their impact on HCP-patient relationships. This research is particularly relevant in the context of Sweden’s robust home care system.

## Methods

### Methodology Overview

This study used qualitative research methods, collecting data through semistructured interviews with HCPs working in advanced home care at Aleris in Sweden. Aleris is one of the major private organizational suppliers of health care in Sweden, offering a range of medical services, including advanced home care and other specialist medical treatments [19]. This approach suited the study’s objective, which aimed to gain a deep understanding of HCPs’ perspectives on using eHealth tools in advanced home care and their impact on relationships with patients. The flexibility of qualitative research allowed nuanced meanings to emerge from participants, captured through direct quotations from interview transcripts. The study adopted an exploratory design to further explain the role of eHealth tools in advanced home care as perceived by HCPs and examined how these tools influence their interactions with patients. This design proved beneficial in uncovering connections between ideas that were either underrepresented or not previously demonstrated in the literature [20]. An inductive approach [21] analyzed the data, aligning with the study’s objectives.

### The eHealth Tools

The eHealth tools used in the study are part of the SwipeCare health care process management system, which partially integrates into TakeCare, the electronic health record (EHR) system used by HCPs in their daily practice. TakeCare serves as the main repository for patient information and is used for routine clinical documentation. SwipeCare functions as an overlay on TakeCare, enhancing the existing EHR system by adding functionalities, such as iPad (Apple Inc)-based questionnaires, calculations, creating and sending questionnaires to patients, and patient reminders [22]. SwipeCare controls and coordinates the patient care process from diagnosis and treatment to aftercare and follow-up. It allows the organizational suppliers of health to define and manage the entire health care process, initiating actions within that process, some performed by SwipeCare itself (like sending out questionnaires) and others by HCPs or other units. SwipeCare interacts with TakeCare only when it needs to read from or write to the patient’s record, thus complementing rather than replacing the EHR.

The 2 tools chosen for the evaluation were a mobile documentation tool and a mobile preconsultation form. The tools were chosen for their complementary nature and potential to capture a wide range of HCP-patient interactions. While the mobile documentation tool was used by the HCP, or collaboratively by HCP and patients, during in-person visits, the mobile preconsultation form was completed independently by patients at home. This diversity in tool use allowed a comprehensive exploration of eHealth’s impact on HCP-patient relationships across different contexts.

The iPad-based mobile documentation tool consists of different predefined standardized forms, which HCPs, either themselves or together with the patient, fill in. Such a form could, for example, cover the required information related to the admission of the patient to a ward or a checklist for evaluating the patient’s physical status after an adjustment in the treatment. The questions in the form consist of predefined categories designed to guide HCPs in documenting patient information. Caregivers have the discretion to determine which answers are available for HCPs to fill in. In some cases, there is a designated “comments” section where HCPs can provide additional explanations or insights. However, in other instances, caregivers may choose to limit responses to predefined alternatives. This limitation mirrored traditional documentation methods, such as pen-and-paper systems, where options were similarly restricted. In addition, certain calculations, such as the Mini Nutritional Assessment (MNA) [23] or the National Early Warning Score 2 (NEWS2) [24], necessitate numerical input and cannot accommodate text responses. This further underscored the importance of predefined answer alternatives within the tool. In addition, the forms ensure no relevant aspects are missed, as questions can be made mandatory to answer.

The second tool, the mobile preconsultation form, is part of the patient portal in the system, which enables the patient to access their own portal, or web page, on their mobile or equivalent digital device. The patient first receives an SMS text message with a link to their patient portal. The patient logs in using a secure method and can then access the preconsultation form. The patient answers the questions at their own pace and time and finally sends the answers back to the caregiver. This enables HCPs to review the status of the patient before traveling to the patient’s home, avoiding the time spent on questioning and filling in answers. Instead, it allows that time to be spent caring for the patient where the patient has its needs, for example, pain in the foot or problems with digestion. The intention is to free HCPs from administration to devote time to the care of the patient and encourage patients to take a more active role in their own care.

### Data Collection and Participant Selection

The recruitment process involved contacting selected participants via email, facilitated by managers from each of the 9 participating clinics in Stockholm, Sweden. Clinics were selected to ensure geographic diversity across the Stockholm region, including clinics from the north, south, and other areas. Clinic heads collaborated in identifying suitable participants with experience using the eHealth tools. This selection strategy aimed to capture a comprehensive range of perspectives, strengthening the validity of the findings. Inclusion criteria for participants comprised individuals aged ≥18 years, officially employed at these clinics, and experienced in using the eHealth tools in their professional roles. The diversity in HCPs’ roles and experience levels allowed for capturing a broad view of eHealth tool use in advanced home care.

Interviews took place between March and May 2023. Participants received the informed consent form via email before the interviews, which they reviewed and signed in person at the beginning of the interview session. Audio recordings and notes documented each 30- to 45-minute interview. A structured interview guide provided consistency while allowing the interviewer flexibility to explore topics relevant to HCPs’ perspectives further. For example, one question from the interview guide asked participants to describe how they adapted their use of eHealth tools based on individual patient needs, providing deeper insights into their experiences and perceptions regarding the patient-HCP relationship.

The characteristics of the 20 HCPs who participated in the interviews varied across the 9 clinics. Each clinic contributed between 1 and 4 HCPs, with a median of 2, ensuring a good spread of perspectives across the clinics involved. Participants were aged between 24 to 59 years, with a mean of 45 (SD 9.3; IQR 41-52) years.

Table 1 presents participants’ characteristics, categorized by profession, years of practice, duration of using mobile documentation tools, and duration of using mobile preconsultation forms. All (20/20, 100%) participants used the mobile documentation tool, while only 60% (12/20) of them used the mobile preconsultation form. This difference in tool use reflects the phased implementation approach common in health care settings, allowing for the gradual adoption and refinement of new technologies. The relatively short duration of eHealth tool use for many participants (0-2 years) illustrates the dynamic nature of health care staffing, characterized by a high turnover rate [25]. HCPs joining from other organizational suppliers of health care or units typically lacked previous experience with these specific eHealth tools. This limited experience duration accurately represents the reality found in many health care units and provides valuable insights into the initial adoption phase of eHealth tools in advanced home care.

These factors—the selection process, varied tool familiarity, and relatively short use duration—shaped the findings by offering a realistic snapshot of eHealth tool adoption in health care units where staff turnover is common. While this scenario limits observations on long-term impacts, the study offers a pragmatic view of eHealth tool implementation, helping stakeholders set realistic expectations and develop strategies that account for the realities of staff turnover and varying levels of tool familiarity in health care settings.

**Table 1:** Participant characteristics.

Participant	Duration of clinical practice^a^ (y)	Duration of the use of mobile documentation tools (y)	Duration of the use of mobile preconsultation form (y)
Nurse 1	6-10	0-2	—^b^
Nurse 2	16-20	0-2	—
Nurse 3	6-10	0-2	0-2
Nurse 4	6-10	0-2	0-2
Nurse 5	6-10	0-2	0-2
Nurse 6	0-5	0-2	0-2
Nurse 7	6-10	3-5	3-5
Nurse 8	0-5	0-2	0-2
Nurse 9	0-5	3-5	3-5
Nurse 10	0-5	0-2	0-2
Nurse 11	6-10	>5	0-2
Nurse 12	11-15	0-2	—
Nurse 13	0-5	0-2	0-2
Nurse 14	6-10	0-2	0-2
Nurse 15	0-5	0-2	0-2
Assistant nurse 1	6-10	0-2	—
Assistant nurse 2	6-10	0-2	—
Assistant nurse 3	0-5	0-2	—
Assistant nurse 4	0-5	3-5	—
Operational therapist 1	>20	0-2	—

^a^Clinical practice: total time spent as health care professionals across all settings, including advanced home care clinics.

^b^Not applicable.

### Data Analysis

The interview, recorded in audio format, underwent verbatim transcription and subsequent review for accuracy and completeness. Following the transcription of the data, participants’ responses were systematically organized and categorized. The analysis was conducted using a content analysis approach grounded in the methodology described by Graneheim and Lundman [26]. The process began with a thorough data familiarization. To gain a deep understanding of its content, we immersed ourselves in the material, reading it carefully multiple times. Once a comprehensive understanding of the data was achieved, we identified *meaning units*—specific segments of text that conveyed key information relevant to the study’s objectives. These meaning units were then condensed to retain their core meaning while eliminating unnecessary details. Each condensed meaning unit was assigned a code, which served as a concise label capturing its essential message. The codes were reviewed and grouped into *subcategories* based on shared patterns and similarities, providing a more detailed and refined understanding of the data. These subcategories were subsequently combined into broader *categories* that represented the distinct dimensions of our study. We then merged related categories and identified key subthemes. Ultimately, we consolidated these findings into an overarching theme. This theme captured the core insights from the data, highlighting both the benefits and challenges of eHealth tool use in advanced home care from HCPs’ perspectives.

### Ethical Considerations

This research was conducted in Sweden. According to the Swedish Ethical Review Act (SFS 2003:460) [27,28], this study does not require ethics approval as it does not involve the handling of sensitive personal information, as defined by the European General Data Protection Regulation (GDPR), Regulation (EU) 2016/679 [29]. However, we acknowledge that ethical principles still apply and have been followed in accordance with relevant regulations and research guidelines.

Prospective study participants received an invitation email outlining the study’s purpose. Those who agreed to participate were contacted via email, SMS text messages, or phone calls to schedule interviews according to their preferences. Before beginning the interviews, participants were provided with a comprehensive explanation of the study objectives, interview procedures, and their rights. Both verbal and written consent were obtained from each participant, with informed consent signed by the participant and the researcher. The consent form included information regarding the aim of the study, potential risks and discomforts, potential benefits, how confidentiality would be handled, the right to withdraw, and consent procedures. It also assured participants of their confidentiality. All participants were informed of their right to withdraw from the study at any stage. Furthermore, no monetary or nonmonetary compensation was provided to any participants. Finally, they were assured that all raw data, comprising audio recordings and interview notes, would be stored on a secured laptop with biometrics log-in (fingerprint) for enhanced data security.

## Results

### Overview of Data Analysis

The data analysis identified one main theme*—optimizing health care with eHealth: enhancing care delivery and overcoming challenges for future health care*. Two subthemes emerged: (1) enhancing care delivery, collaboration, and overcoming adoption barriers and (2) streamlining implementation and advancing eHealth tools for future health care delivery. Five categories were also identified: (1) positive experiences and benefits, (2) interactions between HCPs and patients, (3) challenges and difficulties with eHealth tools, (4) integration into the daily workflow, and (5) future directions. Table 2 shows the subcategories, categories, subthemes, and themes that emerged after coding.

The overarching theme highlighted the study’s central focus on the HCPs’ perspectives on the use and integration of eHealth tools in their daily practice. It emphasized how these tools impact the dynamics of health care delivery, including their influence on the relationships between HCPs and patients, and how they shape the overall patient care experience.

**Table 2:** Overview of the *optimizing health care with electronic health: enhancing care delivery and overcoming challenges for future health care* theme, subthemes, categories, and subcategories.

Subtheme and categories		Subcategories
**Enhancing care delivery, collaboration, and overcoming adoption barriers**		
	Positive experiences and benefits	Efficiency Better documentation and enhanced patient safety
	Interactions between HCPs^a^ and patients	Unaffected HCP-patient relationships Enhanced patient engagement Communication barrier
	Challenges and difficulties with eHealth tools	Technical and resource challenges Patient-related factors
**Streamlining implementation and advancing eHealth tools for future health care delivery**		
	Integration into the daily workflow	Ease of use System integration and application
	Future directions	Staff recommendations Continuity of use

^a^HCP: health care professional.

### Enhancing Care Delivery, Collaboration, and Overcoming Adoption Barriers

#### Positive Experiences and Benefits

The participants highlighted several benefits of using the mobile documentation tool. One significant advantage was the decreased documentation time during patient consultations. The mobile tool allows HCPs to document directly during patient encounters, which increase *efficiency* by eliminating the need for double documentation [30] and reducing the time spent on paperwork. Approximately two-thirds (13/20, 65%) of the HCPs indicated that the mobile documentation tool helped them save time on documentation:

I think it [the mobile documentation tool] decreases my work time [taking clinical notes during consultation].

Nurse 6

It saves me time because I document while I am at the patient’s house.

Nurse 9

Another benefit mentioned was the standardization of content for clinical note-taking. The use of standardized templates within the mobile tool ensured that all relevant information was captured consistently, which is crucial for effective patient care. Participants also expressed an enhanced sense of safety and security for patients when using the mobile documentation tool. This improvement was attributed to more accurate and timely documentation of health status, which contributed to better patient outcomes. The time saved through efficient documentation potentially translated to improved patient care quality. With reduced administrative burden, HCPs could devote more time to meaningful patient interactions, allowing more in-depth discussions about health concerns and treatment plans.

While most (13/20, 65%) participants perceived the mobile documentation tool as beneficial, some noted challenges. A few (7/20, 35%) participants reported that it disrupted their routine workflow by prolonging consultation times. They found that carrying the iPad during home care visits added to their equipment load, making it inconvenient:

I think it [the mobile documentation tool] increases my work time. My usual consultation is about 20-30 minutes, with it [the mobile documentation tool], it can take 45-50 minutes. We carry a lot of equipment during home care visits, including a laptop, and it can be heavy. The iPad is an additional weight.

Nurse 1

Because we cannot do everything we need in the [mobile documentation] tool, it kind of doubles the work for us.

Nurse 4

Among users of the mobile preconsultation form, participants highlighted several advantages, including knowing the patient’s medical concerns in advance and saving time on preparations. This previsit work done by patients reduced the administrative workload on HCPs, allowing for a more efficient use of consultation time.

Standardizing documentation through the mobile documentation tool and preconsultation form may lead to more consistent and thorough patient assessments across different HCPs. This standardization ensures that all relevant information is captured systematically, potentially improving the continuity of care and reducing the risk of overlooking critical patient data.

The use of eHealth tools in advanced home care led to *better documentation practices while enhancing patient safety*. The majority (13/20, 65%) of the participants found the standardized content of the mobile documentation tool beneficial, noting that it ensured they would not overlook critical information:

It is kind of nice that the [mobile documentation] tool has a lot of information that can help with taking notes. Also, the setup is the same for everyone, so I think the note-taking is sort of standardized.

Nurse 1

This standardized approach to documentation not only improved consistency across different HCPs but also potentially enhanced the quality of patient care. By ensuring comprehensive data collection, the tool might lead to more informed clinical decision-making and improved continuity of care. The structured format guided HCPs through specific assessment points, helping to ensure that all relevant aspects of patient care were addressed during consultations. Improved documentation through eHealth tools contributed to patient safety and security. Participants noted that accurate information recorded in EHRs reassures patients, helping them feel more secure in their care:

When we use [the mobile documentation tool], we can check the necessary things we have to ask the patient. We will not forget anything because we have a guide, I think that is good for the patient...it can increase patient security.

Nurse 13

I think they [patients] feel safer when I can write everything in the [mobile documentation] tool while I am with them, and they can check what I write.

Nurse 8

The ability for patients to review clinical notes during consultations further enhanced their confidence, fostering a sense of transparency in the health care process and further strengthening the relationship. This aligned with the principle of “equal care” in Sweden, which emphasizes the importance of providing consistent quality care to all patients, regardless of their location or the HCPs they encounter [31].

By standardizing documentation practices, eHealth tools contributed to this principle by ensuring that all relevant patient information is consistently captured and accessible to different HCPs. This standardization not only improved continuity of care but also enhanced trust between patients and HCPs. The structured format of the mobile documentation tool facilitated comprehensive data collection, allowing HCPs to focus on critical issues during consultations. This approach optimized patient interactions and strengthened the overall quality of care provided, aligning with Sweden’s commitment to equitable health care access.

#### Interactions Between HCPs and Patients

All (20/20, 100%) participants indicated that their *relationship with patients remained unaffected* by the implementation of both the mobile documentation tool and the mobile preconsultation form. This finding suggested that HCPs were able to maintain their interpersonal skills and patient-centered approach while integrating eHealth tools into their practice:

I think nothing has changed in how I interact with patients when using it [the mobile documentation tool].

Nurse 5

The consistency in relationships despite introducing new technology highlighted the adaptability of HCPs in maintaining effective communication with patients. While the eHealth tools introduced new elements to patient interactions, such as the physical presence of devices, HCPs appeared to successfully navigate these changes without compromising the quality of their patient relationships. Furthermore, the mobile preconsultation form may have enhanced patient engagement by allowing patients to provide more comprehensive information before consultations. This previsit work could potentially lead to more focused and in-depth discussions during face-to-face interactions, without negatively impacting the HCP-patient relationship.

The mobile preconsultation form emerged as a tool that potentially *enhances patient engagement* in their care. Some (3/12, 25%) participants noted that certain patients provided more comprehensive information through written responses on the mobile preconsultation form than during face-to-face interactions with the HCPs. This suggested that the tool may lead to more informed and collaborative interactions during consultations:

If you send out the form, I think you can get more information from the patients because they [patients] have more time to think about it rather than when we ask them in person.

Nurse 7

The previsit work done by patients allowed them to elaborate on both current problems and positive aspects of their health status. This more comprehensive written information might shift the dynamics of face-to-face interactions, potentially allowing more focused and in-depth discussions during consultations. Furthermore, the mobile preconsultation form’s ability to highlight patients’ medical concerns in advance enabled HCPs to prepare more effectively for consultations. This preparation may contribute to more efficient and patient-centered interactions, as evidenced by one participant’s comment:

We send out the form once every four weeks for when we do the team rounds, it can help us in doing the team rounds, making it faster and more efficient, in my experience.

Nurse 5

By facilitating more comprehensive information gathering and allowing HCPs to focus on critical issues, the mobile preconsultation form may enhance patient engagement and potentially improve the quality of HCP-patient interactions in advanced home care settings.

Participants mentioned *communication barrier* as a challenge. While the eHealth tools offered numerous benefits, 20% (4/20) of the participants observed noted challenges in their use during patient interactions. These HCPs noted that the iPad (mobile documentation tool) could sometimes be a barrier to effective communication:

When I am holding the iPad in front of the patient, it kind of becomes a barrier between us.

Nurse 6

When using the iPad, you tend to look down instead of looking at the patient directly.

Nurse 9

Maybe some patients find it rude [using an iPad during consultation], but most of them understand that we do it to get everything right. Also, I think sometimes I have a less open conversation with patients.

Nurse 10

Most (16/20, 80%) participants did not report communication issues, suggesting that the majority of HCPs were able to integrate the eHealth tools without significant impact on patient interactions. Compared to traditional pen-and-paper methods, the eHealth tool presented both advantages and challenges for patient-HCP communication. While pen-and-paper notes may feel less intrusive during patient interactions, the eHealth tool offers improved legibility, standardization, and ease of data retrieval, potentially enhancing the quality of information shared with patients. However, the physical presence of the device could create a perceived barrier for some HCPs and patients. The eHealth tool’s structured format ensures comprehensive documentation, potentially reducing the risk of omitting crucial information compared to freeform paper notes. This standardization may lead to more consistent and thorough patient assessments across different HCPs, indirectly benefiting patient care and communication. However, this structure might sometimes limit the flexibility in capturing nuanced patient narratives.

Regarding focus on issues and medical decision-making, the eHealth tool’s ability to provide instant access to patient history and standardized assessments can potentially enhance HCP’s ability to focus on critical issues during patient interactions. However, as noted by some participants, the tool might occasionally divert attention from direct patient interaction. These observations highlighted the need for balanced use of eHealth tools, combining their benefits with maintaining effective patient communication. HCPs may need to develop strategies to integrate these tools seamlessly into their patient interactions, ensuring that technology enhances rather than hinders the HCP-patient relationship.

#### Challenges and Difficulties With the eHealth Tools

While most (14/20, 70%) participants reported no technical issues, some HCPs encountered *technical and resource challenges* when using eHealth tools in advanced home care. Specifically, 30% (6/20) of the participants faced technical problems with the mobile documentation tool, including log-in difficulties, frequent software updates, and issues importing clinical notes to the patient’s EHR:

First, when you come in to work, you have to log in to the [mobile documentation tool]. Sometimes, it’s a problem. Now, with the frequent updates—they do updates like every week—there is often something wrong after each update...

Nurse 3

Notably, 100% (12/12) of the participants who used the mobile preconsultation form reported no technical issues at all. Resource limitations were a more widespread concern. All (20/20, 100%) participants noted the need to share iPads among staff members, potentially limiting access and flexibility in documentation:

The staff has to share iPads [with the mobile documentation tool] within the team.

Nurse 9

These challenges highlighted the importance of robust IT infrastructure and adequate resource allocation in eHealth implementation. While technical issues affected only a minority (6/20, 30%) of users, device sharing impacted all (20/20, 100%) participants, potentially influencing HCP efficiency and, consequently, patient care quality.

*Patient-related factors*, such as patient characteristics, influenced the adoption and use of eHealth tools in advanced home care. Approximately 55% (11/20) of the participants noted that the patient’s age and health conditions affected receptiveness to eHealth tools, particularly the mobile preconsultation form:

Not all patients are open to using the [mobile preconsultation] form. Usually, older patients or those with difficult diseases are not open to it.

Nurse 12

HCPs demonstrated adaptability by adjusting their approach based on patient characteristics. This flexibility was crucial for maintaining quality care across diverse patient populations. However, it also raised concerns about potential disparities in care delivery between technologically proficient patients and those less comfortable with eHealth tools. Some patients expressed skepticism about using the mobile preconsultation form, particularly due to privacy concerns:

There are patients who are skeptical about using it [the mobile preconsultation form], as it involves using their [personal information] BankID [a Swedish electronic identification system].

Nurse 11

On the other hand, a subset of patients, predominantly younger individuals, demonstrated an openness to using eHealth tools, according to 55% (11/20) of the participants. This varying receptiveness highlighted the need for a personalized approach in implementing eHealth tools in advanced home care settings, ensuring that all patients receive appropriate care regardless of their technological proficiency.

### Streamlining Implementation and Advancing eHealth Tools for Future Health Care Delivery

#### Integration Into the Daily Workflow

Regarding *ease of use*, 75% (15/20) of the participants found the mobile documentation tool easy to use. The initial introduction and demonstration of these eHealth tools to HCPs lasted an hour and was perceived as sufficient by all the 12 (12/12, 100%) participants who received it. Some (4/20, 20%) participants had the opportunity to familiarize themselves with the tool beforehand as part of the implementation team, while 12 (12/20, 60%) participants received information during introductory seminars at their clinics. Some (4/20, 20%) participants only underwent introductory training after using the mobile documentation tool:

I have tried it [the mobile documentation tool], honestly, without any training... I tried to use it... I think it is easy.

Assistant Nurse 2

My colleague taught me how to use it [the mobile documentation tool].

Nurse 8

In addition, 25% (5/20) of the participants reported that the log-in process and navigating through all the components of the mobile documentation tool required some time to become familiar with, but they gradually improved with more use:

As I continue to use it [the mobile documentation tool], I learn more about it, which makes me better at using it.

Nurse 2

Regarding *system integration and adaption* all (20/20, 100%) participants highlighted challenges in integrating eHealth tools into existing systems and their practical *application* in advanced home care. A key limitation was the incomplete integration of the mobile documentation tool with the existing EHR system, TakeCare. While clinical notes could be uploaded to the patient’s journal, other crucial data remained inaccessible through the mobile tool, requiring staff to rely on computers for a complete view of patient information:

It [the mobile documentation tool] doesn’t have all the functions that you have in the EHR.

Nurse 7

[B]ecause with the computer, I can do and see everything on the patient’s EHR, but not with the iPad.

Nurse 4

The lack of full system integration disrupted seamless workflows and raised concerns about potential inconsistencies in documentation and delays in accessing critical information during consultations. Participants expressed a strong desire for improved interoperability to ensure that all necessary data are accessible on the mobile device.

Regarding practical application, HCPs generally found the mobile preconsultation form useful for integrating into their existing workflows. However, its applicability was perceived as limited for certain patient groups and care scenarios. For example, HCPs noted that sending the form before every consultation was unnecessary for patients with chronic conditions requiring long-term care as their primary clinical concerns often remained consistent:

For example, I have had this patient for a very long time, and the patient has a chronic condition. I don’t need to send the form [before] every visit because the status is still the same.

Nurse 11

Despite these limitations, participants acknowledged the value of the preconsultation form for new patients or those with changing health conditions. It allowed HCPs to gather detailed information in advance, facilitating more focused consultations. These findings underscored the importance of improving system integration to enhance workflow efficiency while adapting eHealth tools to meet diverse patient needs in advanced home care settings.

#### Future Directions

HCPs shared *recommendations* on enhancing the effectiveness of the eHealth tools in their daily workflow in advanced home care. Most (16/20, 80%) participants expressed that completely integrating the mobile documentation tool with the patient’s EHR system would improve efficiency. They suggested that accessing the same information as in the patient’s EHR directly on the mobile device would eliminate the need to switch between the iPad and the computer for clinical documentation, improving their workflow:

Lab results and checking off the to-do list, that is what is still missing, I think...and an easier login.

Nurse 9

All (12/12, 100%) HCPs who used the mobile preconsultation form found it adequate. However, 17% (2/12) of the participants suggested further enhancements to the interface, including languages other than Swedish:

The [mobile preconsultation] form does not look very good, in my opinion. It can be better.

Nurse 11

Regarding equipment use, half (10/20, 50%) of the participants expressed that having sufficient iPads for all staff members would be advantageous, reducing the need for frequent logging in and out.

Approximately two-thirds (14/20, 70%) of participants anticipated *continuity of use* of the 2 eHealth tools in advanced home care, acknowledging the emergence of the new technologies. In addition, 45% (9/20) of the HCPs expressed optimism regarding the increased patient acceptance of technology in health care:

I think they [patients] will use them [eHealth tools] more and more in the future.

Nurse 7

## Discussion

### Principal Findings

The aim of this study was to explore HCPs’ perspectives on using eHealth tools in advanced home care and their impact on HCP-patient relationships. In total, 20 HCPs from 9 different advanced home care clinics participated in the interviews, leading to the identification of five categories: (1) positive experiences and benefits, (2) interactions between HCPs and patients, (3) challenges and difficulties with the eHealth tool, (4) integration into the daily workflow, and (5) future directions.

Overall, most (13/20, 65%) HCPs expressed a positive perspective regarding the 2 eHealth tools used in the study: the mobile documentation tool and the mobile preconsultation form. The results showed that HCPs identified several benefits for themselves and their patients. Notably, 65% (13/20) of the participants reported that the mobile documentation tool allowed them to document directly during patient encounters, eliminating double documentation and saving time. Standardized templates within the tool ensured consistency in clinical note-taking, which participants believed enhanced patient safety by reducing errors and omissions.

While most (13/20, 65%) HCPs reported increased efficiency, 35% (7/20) experienced the opposite, suggesting that user acceptance of eHealth is multifaceted. These participants reported that the mobile documentation tool disrupted their routine workflow by prolonging consultation times and adding to their equipment load during home care visits. This highlights the importance of considering individual user experiences in eHealth implementation. The ease of use of the eHealth tools was a significant factor in their integration into daily workflows. Most (15/20, 75%) of the participants found the mobile documentation tool easy to use, indicating successful integration. This high level of usability likely contributed to the overall positive reception of the tools and suggested that well-designed eHealth solutions can be effectively adopted in advanced home care settings.

However, HCPs noted challenges, both tool-related and patient-related. Some (6/20, 30%) of the participants faced technical problems with the mobile documentation tool, including log-in difficulties and issues importing clinical notes to the EHR. These technical challenges highlight the need for robust IT infrastructure and ongoing technical support in eHealth implementation. Resource limitations were a widespread concern, with all participants noting the need to share iPads among staff members. This sharing potentially limits access and flexibility in documentation, impacting workflow efficiency. Future eHealth implementations should consider resource allocation to ensure adequate device availability for all HCPs.

Patient characteristics influenced the adoption of eHealth tools, particularly the mobile preconsultation form. Approximately 55% (11/20) of the participants noted that patient age and health conditions affected receptiveness to these tools. This finding underscores the importance of tailoring eHealth implementations to diverse patient populations and considering alternative approaches for patients less comfortable with technology. The mobile preconsultation form emerged as a tool that potentially enhances patient engagement. Some (3/12, 25%) of the participants using this tool noted that certain patients provide more comprehensive information through written responses than face-to-face interactions with HCPs. This previsit work allows for more focused and efficient consultations, potentially improving the quality of HCP-patient interactions.

Regarding the HCP-patient relationship, all (20/20, 100%) participants reported that the use of eHealth tools had no significant impact on the relationship with patients. This suggests that HCPs successfully maintained their interpersonal skills and patient-centered approach despite introducing new technology. However, 20% (4/20) of the participants noted that using devices such as iPads could occasionally act as a communication barrier during consultations, highlighting the need for strategies to seamlessly integrate technology into patient interactions.

Looking to the future, 70% (14/20) of the participants anticipated continued use of the eHealth tools in advanced home care, reflecting their perceived long-term value despite existing challenges. In addition, 45% (9/20) of the HCPs expressed optimism about increased patient acceptance of technology in health care. While participants did not report changes in HCP-patient interactions, they emphasized that current eHealth tools successfully maintain existing relationships without negatively impacting communication or rapport.

### Comparison With Prior Work

Mathijssen et al [32] highlighted a similar finding of positive perception of eHealth by HCPs in home care. Despite recognized challenges such as inadequate training and support, data privacy concerns, and the necessity for more user-friendly technology, eHealth in home care is seen as a promise to enhance care quality. A study focusing on a specific eHealth tool, electronic medication administration records, emphasized the importance of cautious implementation due to potential unintended consequences [33]. Participants noted that the electronic medication administration records introduced new tasks alongside existing nursing tasks. In contrast, our study found no perceived changes in the workflow or work situation due to eHealth tools, potentially contributing to their overall positive reception.

Carlqvist et al [34] conducted a qualitative interview study examining how an eHealth application can serve as a value-creating resource from the perspective of HCPs. Their findings indicated that while such applications can enhance proactive communication and support patient engagement in self-care, challenges remain. Notably, patients’ difficulties in using the application or performing measurements sometimes led to value destruction, requiring time-consuming recovery efforts by professionals. This contrasts with our study, where HCPs reported no significant workflow disruptions due to eHealth tools, potentially contributing to their positive acceptance.

Nakrem et al [35] explored the introduction of digital medicine dispensers in home health care services and their influence on patient-caregiver relationships. They found that while such technologies can improve efficiency and enhance patient independence, they may also strain patient-caregiver relationships if not aligned with patients’ needs and safety concerns. However, in our study, HCPs did not perceive eHealth tools as affecting their relationships with patients, suggesting that the specific type of technology and its implementation context play crucial roles in acceptance and impact.

Furthermore, Ramachandran et al [36] conducted a review examining the impact of eHealth on patient-HCP and HCP-HCP relationships in primary care. They found that eHealth can have both positive and negative effects on relationships and trust, influenced by factors, such as technology design, patient demographics, and organizational implementation strategies. This aligns with our findings, where the successful integration of eHealth tools without perceived workflow disruptions may be attributed to effective implementation strategies and user-centered design.

### Future Research

Future studies could explore strategies for seamlessly integrating eHealth tools into patient interactions without compromising communication quality. In addition, research into personalized eHealth solutions that accommodate diverse patient needs and preferences could address challenges related to patient characteristics. Investigating the long-term impacts of eHealth tools on care quality, patient outcomes, HCP-patient relationships, and HCP job satisfaction would provide valuable insights for future implementations.

Another important area for investigation is the development and implementation of features that actively enhance HCP-patient relationships in advanced home care environments. For instance, improving patient education modules or creating shared decision-making tools tailored to home care settings could address some of the gaps identified in this study, such as maintaining meaningful interactions while using eHealth tools.

Furthermore, exploring the role of relatives in supporting patients with eHealth tools could offer new avenues for enhancing patient engagement and care delivery [37]. Relatives could assist with completing preconsultation forms or managing digital tools for patients who face challenges due to age or health conditions. Recognizing relatives as key stakeholders alongside HCPs may improve overall patient support in advanced home care settings [38].

### Limitations

This study focused specifically on advanced home care settings in Sweden, which may limit the applicability of findings to other health care contexts. The unique characteristics of advanced home care, such as the need for mobile documentation and remote patient monitoring, shaped the experiences reported by HCPs. In addition, the exclusive focus on HCPs’ interviews represents another limitation. Incorporating patients’ perspectives could offer valuable insights and contribute to a more comprehensive understanding of the implementation of eHealth tools in advanced home care.

### Conclusions

HCPs in advanced home care recognize the value of eHealth tools in their daily work while maintaining the quality of their relationships with patients. The study provides insights into both the experiences of HCPs using eHealth tools and how these tools influence their interactions with patients in advanced home care settings. Positive experiences related to eHealth use and the level of integration of eHealth tools into daily work motivate staff to use these tools, potentially allowing for more time and focus on patient interactions. By contrast, negative experiences with the eHealth tools limit their use and acceptance.

Notably, HCPs reported no perceived changes in their relationships with patients when using the mobile documentation tool and the preconsultation form tool. This suggests that eHealth tools can be integrated into care delivery without compromising the personal aspect of HCP-patient interactions. Although most of the staff in the study had a positive view of their use of the eHealth tools, understanding the challenges they encounter is essential to increase acceptance and success in implementation further. Future development should focus on features that not only improve efficiency but also actively enhance HCP-patient relationships in the advanced home care setting.

## Abbreviations

EHR: electronic health record
HCP: health care professional
MNA: Mini Nutritional Assessment
NEWS2: National Early Warning Score 2
